# Current Status of Long Non-Coding RNAs in Human Cancer with Specific Focus on Colorectal Cancer

**DOI:** 10.3390/ijms150813993

**Published:** 2014-08-12

**Authors:** Maria Smolle, Stefan Uranitsch, Armin Gerger, Martin Pichler, Johannes Haybaeck

**Affiliations:** 1 Institute of Pathology, Medical University Graz, Auenbruggerplatz 25, A-8036 Graz, Austria; E-Mail: maria.smolle@stud.medunigraz.at; 2 Department of Surgery, St John of God Hospital Graz, Marschallgasse 12, A-8020 Graz, Austria; E-Mail: Stefan.Uranitsch@bbgraz.at; 3 Division of Oncology, Medical University Graz, Auenbruggerplatz 15, A-8036 Graz, Austria; E-Mails: armin.gerger@medunigraz.at (A.G.); martin.pichler@medunigraz.at (M.P.)

**Keywords:** colorectal cancer, lncRNA, cancer, molecular tumor pathogenesis

## Abstract

The latest investigations of long non-coding RNAs (lncRNAs) have revealed their important role in human cancers. LncRNAs are larger than 200 nucleotides in length and fulfill their cellular purpose without being translated into proteins. Though the molecular functions of some lncRNAs have been elucidated, there is still a high number of lncRNAs with unknown or controversial functions. In this review, we provide an overview of different lncRNAs and their role in human cancers. In particular, we emphasize their importance in tumorigenesis of colorectal cancer, the third most common cancer worldwide.

## 1. Introduction

After the sequencing of the human genome was finished in 2003, it became evident that only 20,000 genes are protein-coding, while over 98% of all genes remain untranslated, giving rise to non-protein-coding RNAs (ncRNAs) [1]. Over the past decade, thousands of ncRNAs were identified in the eukaryotic transcriptome [2,3]. Traditionally, ncRNAs are divided into two groups according to their length: the short ncRNAs, such as microRNA (miRNA) and transfer RNA (tRNA), consisting of less than 200 nucleotides, and the long non-coding RNAs (lncRNAs), sized from 200 nucleotides up to 100 kb [4]. Members of this second group are *Xist* (X-inactive-specific transcript), playing an important role in X-chromosome inactivation, HOTAIR (homeobox gene antisense intergenic RNA), and the imprinted H19—one of the first lncRNA genes being reported [5,6,7,8].

Most lncRNAs are transcribed by RNA polymerase II, but some by RNA polymerase III [9]. While functional lncRNAs are usually polyadenylated many of them remain un-polyadenylated, such as BC200 and Occt4-pg5 [10,11].

### 1.1. Functions of LncRNAs

Presenting a heterogeneous group, lncRNAs play an important role in various biological processes, although most of them are poorly conserved [12,13].

Wang *et al.* [14] subdivided the molecular functions of lncRNAs into four archetypes: signaling, decoying, scaffolding and guiding. The advantage of using lncRNAs for regulative functions lies in avoiding time-consuming translation into proteins and some signaling lncRNAs exhibit regulatory transcriptionally relevant functions [14]. Decoying ncRNAs mainly have a negative effect on regulation by capturing and eliminating RNA-binding proteins (RBPs) which commonly are specific transcription factors [14]. As guides, lncRNAs can either change expression of adjacent genes (*cis*-acting guides), such as *Xist*, *Air* and *COLDAIR*, or, though less frequently, they control expression of distant genes (*trans*-acting guides), like *HOTAIR* and *Jpx* [14,15,16]. The fourth class of scaffolding lncRNAs bring together effectors with activating or repressive impact on transcription by binding them with several lncRNA-domains [14].

Vikram *et al.* [17] published another classification of lncRNAs depending on their location regarding their respective gene. They also devided lncRNAs in four groups: sense, antisense, intronic and intergenic lncRNAs. Sense lncRNAs are transcribed from the same strand of DNA as protein-coding genes, antisense lncRNAs from the opposite strand. In case of intronic lncRNAs the transcription unit lies within an intron of another gene whereas the region for transcriptation for intergenic lncRNAs lies outside of the coding region.

According to Pandolfi and colleagues [18] lncRNAs also bind to microRNA-response-elements (MREs) and do not only interact with other lncRNAs. In diverse experiments, competition for miRNAs could be revealed as an important factor for gene regulation of both lncRNAs and messengerRNAs [19,20,21,22].

### 1.2. LncRNAs in Cancer

Tumor cells proliferate autonomously, are insensitive for stimuli from the organism and produce factors enhancing cell division, avoiding apoptosis and stimulating vascular growth.

Hundreds of oncogenes and tumor suppressor-genes have been discovered, but many cellular mechanisms in tumors are still poorly understood [23]. Recently, the emphasis of researchers concerning tumorigenesis has switched to lncRNAs, as those “dark matters of the genome” are selectively over- or underexpressed in different tumors, as summarized in Table 1 [24].

**Table 1:** Cancer and lncRNA expression.

Type of Cancer	lncRNA		Expression	Ref.
*Head- and Neck Cancer*				
nasopharyngeal carcinoma	LINC00312		down	[25]
*Brain Tumors*				
glioblastoma multiforme	ASLNC22381		up	[26]
	ASLNC20819		up	[26]
Neuroblastoma	ncRAN		up	[27,28]
*Lung Cancer*				
non-small cell lung cancer	TUG1		down	[29]
lung cancer	MALAT-1		up	[30]
*Soft-Tissue Sarcoma*				
Osteosarcoma	TUG1		up	[29,31]
*Tumors of the Genitourinary Tract*				
urothelial carcinoma	ncRAN		up	[32]
	UCA-1		up	[33]
prostate cancer	PlncRNA-1		up	[34]
	MALAT-1		up	[35]
	PCGEM1		up	[36]
	PRNCR1		up	[36]
	PCAT-1		up	[37]
epithelial ovarian cancer	HOTAIR		up	[38]
	UCA-1		up	[39]
	MALAT-1		down	[39]
	H19		down	[39]
*Tumors of the Gastrointestinal Tract*				
esophagus squamous cell carcinoma	PlncRNA-1		up	[40]
	HOTAIR		up	[41]
gastric cancer	HOTAIR		up	[42]
	CCAT1-L		up	[43]
	H19		up	[44]
hepatocellular carcinoma	H19		up	[45]
	MALAT-1		up	[30]
	HOTAIR		up	[46]
	HULC		up	[47,48]
	LncRNA-Dreh		Down	[49]
	MEG3		Down	[50]
	lncRNA-MVIH		up	[51]
	MDIG		up	[52]
*Colorectal Cancer*				
	PCAT-1		up	[53]
	PRNCR1		up	[54]
	MALAT-1		up	[55]
	CCAT1-L		up	[56,57]
	PVT-1		up	[58]
	ncRAN		down	[27,58]
	HOTAIR		up	[59]

LncRNAs acting like oncogenes, are up-regulated in tumor tissue. The metastasis-associated lung adenocarcinoma transcript 1 (MALAT-1) for example, which is expressed in human tissue, becomes up-regulated in various solid tumors, including liver, lung, breast and prostate cancer [30,60].

Some lncRNAs act like tumor-suppressors, such as the maternally expressed gene 3 (MEG3) which is commonly expressed in normal human tissue. However, its down-regulation in most human meningiomas [61] promotes an increase in the expression level of tumor suppressor-protein p53, and the transcription of p53-related, driving defective cells into apoptosis. However, MEG3 also inhibits dysregulated cell proliferation in the absence of p53, leading to the opinion that this lncRNA has a suppressive function in both, p53-dependent and -independent ways [62]. The next subchapters will describe role of lncRNAs in particular types of human cancers in more detail with a special focus on lncRNAs on colorectal cancer.

#### 1.2.1. Head- and Neck Cancer

Nasopharyngeal Carcinoma

In nasopharyngeal carcinoma expression of LINC00312, an intergenic lncRNA with tumor-suppressive function, is significantly down-regulated [25]. Under physiological conditions LINC00312 inhibits proliferation in nasopharyngeal epithelium by preventing cell cycle passage from the G1 into S phase but increases cell adhesion, motility and invasion by down-regulating the expression of estrogene receptor alpha (ERα) [63]. A lower expression level of LINC00312 in nasopharyngeal tumor tissue is positively correlated with tumor size and clinical stage but negatively correlated with lymph node metastasis [25].

#### 1.2.2. Brain Tumors

Glioblastoma Multiforme

In glioblastoma multiforme (GBM), 654 lncRNAs were shown to be up-regulated and 654 down-regulated at least fourfold in comparison to normal brain tissue [64]. Two up-regulated lncRNAs, ASLNC22381 and ASLNC20819, might play important roles in malignant behavior and relapse of GBM by targeting insulin-like growth factor (IGF-1) genes [26].

#### 1.2.3. Lung Cancer

Non-Small Cell Lung Cancer

The lncRNA taurine-upregulated gene 1 (TUG1) is down-regulated in over 80% of non-small cell lung cancers (NSCLC; squamous cell carcinoma, adenocarcinoma) compared to normal tissue which is positively correlated with tumor size and pathological stage [29]. 

TUG1-expression is regulated by binding of p53 to a response element at the promotor region [29]. TUG1 epigenetically regulates the expression of HOXB7, an oncogene activating the AKT/MAPK (protein kinase B/mitogen activated protein kinase) pathways [65].

#### 1.2.4. Soft-Tissue Sarcoma

Osteosarcoma

In osteosarcoma TUG1 usually is overexpressed causing high cell proliferation and a low apoptosis rate, whereas in other cancer tissues TUG1 is down-regulated [29,31]. This emphasizes the theory that lncRNAs are expressed in a more tissue-specific pattern than protein-coding RNAs, a feature that makes them attractive as potential diagnostic biomarkers [40,41].

#### 1.2.5. Tumors of the Genitourinary Tract

Urothelial Carcinoma/Bladder Cancer

The long non-coding RNA urothelial carcinoma associated 1 (lncRNA-UCA1), a key player in cancer progression and metastasis formation of urinary bladder cancer, was shown to be up-regulated in bladder cancer [33]. However, the pathways contributing to its function are largely unknown. Xue *et al.* [66] identified a CCAAT/enhancer binding protein α (C/EBPα), which is interacting with lncRNA-UCA1 in cancer cells. C/EBPα bound to the core promoter region *in vitro* and *in vivo* [66]. Upon treatment with C/EBPα siRNA the expression of lncRNA-UCA1 was depressed, cell viability was reduced and apoptosis induced *in vitro*. Due to its high sensitivity and specificity, lncRNA-UCA1 can be used as a biomarker to detect bladder carcinoma in urine sediment [33]. Based on these data, lncRNA-UCA1 might also serve as a therapeutic target once the appropriate delivery systems and tools for targeting lncRNAs at specific locations will be developed.

*UCA1* has three variant transcripts which were detected by northern blot analysis in the human bladder transitional cell carcinoma (TCC) cell line BLZ-211 by Wang and colleagues [67]. This group focused on the second transcript (2.2 kb) (UCA1a) which is identical to the cancer up-regulated drug resistant gene (*CURD*). Overexpression of *UCA1a* (*CURD*) was reported to lead to increased proliferation, migration and invasion of urinary bladder cancer cells. The increased apoptosis rate by cisplatin in UM-UC-2 cells was antagonized by the overexpression of CURD.

Zhu *et al.* [32] investigated the behavior of ncRAN, an lncRNA, in urinary bladder cancer cell lines. The expression was significantly higher in invasive tumor cells compared to normal tissue. An overexpression in the superficial tumor cell line (RT4) promoted cell proliferation, migration and invasion.

Prostate Cancer

The influence of lncRNAs on the androgen receptor is a point of interest due to the fact that the androgen receptor is a transcription factor and plays a key role in growth and survival of prostate cells. Therapeutic blocking of androgen receptors (ARs) leads to tumor regression in prostate cancer. The two lncRNAs prostate cancer noncoding RNA1 (PRNCR1) and prostate cancer gene expression marker 1 (PCGEM1) were reported by Yang [36] to bind to ARs and mediate gene transcription. They are overexpressed in aggressive prostate cancer and activate both ligand-dependent and ligand-independent AR-mediated proliferation. However, the association of PNCR1 and prostate cancer could not be replicated by others [68]. Ren *et al.* [35] investigated MALAT-1 in human prostate cancer tissue and in mouse xenografts and showed that MALAT-1 was upregulated in both. This was correlated with markers of poor prognosis, such as a high gleason score, prostate-specific antigen, tumor stage and castration-resistant prostate cancer. Silencing of MALAT-1 by siRNA transfection inhibited cell growth, invasion and migration and induced cell cycle arrest in the G0/G1 phase in castration resistant prostate cancer.

LncRNA PCAT-1 was investigated in prostate cancer (PC) by Presner *et al.* [69]. PCAT-1, located on chromosome 8q24, was reported to be upregulated in metastatic and localized PC. Polycomb Repressive Complex 2 (PRC2) is interacting with PCAT-1 to promote cell proliferation. PCAT*-*1 modulates the transcriptional regulation of 370 genes, 255 of which are upregulated, leading to an increased cell turnover. PCAT-1 represses the BRCA2 tumor suppressor gene which in consequence leads to less homologous recombination (HR). A mutation of *BRCA2* leads to defective HR and accumulation of double-stranded DNA breaks [70]. High expression of PCAT-1 in PC tissue was reported to predict for low *BRCA2* expression [69].

Epithelial Ovarian Cancer 

The expression levels and clinical relevance of lncRNA HOTAIR in epithelial ovarian cancer (EOC) was investigated by Qui *et al.* [38]. They used surgically resected tissue samples of ovarian tumors, non-cancerous ovarian surface epithelial tissue and human ovarian cancer cell lines (SKOV3, SKOV3.ip1, HO8910, HO8910-PM and HEY-A8). The expression of HOTAIR was measured by qRT-PCR (real time reverse transcription polimerase chain reaction) and was significantly higher in the tumors than in normal epithelial ovarian tissue. Analyzing the clinical relevance of high HOTAIR levels in EOC a positive correlation with an advanced FIGO (International Federation of Gynecology and Obstetrics) stage, high histological grade of the tumor and occurrence of lymph node metastases was demonstrated [38]. The overexpression of HOTAIR was found to be independently associated with shorter disease free survival and poor overall survival of the patients [38].

A knockdown of *HOTAIR* in cell lines and in a mouse model *in vivo* was reported to inhibit migration and invasion in SKOV3.ip1, HO8910-PM, and HEY-A8 cells. These findings suggest that HOTAIR promotes EOC metastasis [38].

The human ovarian cancer cell lines SKOV3 and SKOV3.ip1 were used to determine the difference in the expression of lncRNAs in EOC by using a microarray approach. The SKOV3.ip1 cells had a higher metastatic potential than the other cell line [71]. Liu *et al.* [39] reported H19, LOC100292680 and MALAT-1 as down-regulated, UCA1, CCAT1*,* LOC645249, and LOC100128881 as up-regulated in SKOV3.ip1 cells. 

#### 1.2.6. Tumors of the Gastrointestinal Tract

Esophageal Squamous Cell Carcinoma

According to Wang *et al.* [34], PlncRNA-1 is up-regulated in esophageal squamous cell carcinoma (ESCC), the eighth most common cancer worldwide. In ESCCs with lymph node metastases PlncRNA-1 expression was higher than in carcinomas limited to the esophagus. Moreover, *in vitro* knock-down of PlncRNA-1 increased the apoptosis rate in tumor tissue significantly pointing to a suppressive effect of this lncRNA on controlled cell death [34].

Furthermore, the expression of the oncogenic lncRNA HOTAIR is elevated in ESCC in comparison to noncancerous tissue which indicates a poor prognosis [72].

Gastric Cancer

Wang *et al.* [73] analyzed the expression of lncRNAs in gastric adenocarcinoma (GCA) and compared the results to normal tissue. One-thousand, eight-hundred and eighty lncRNAs were significantly up-regulated, 294 were down-regulated. The most up-regulated lncRNA was ASHG19A3A028863 (fold change: 146.0139123) and the most down-regulated lncRNA ASHG19A3A007184 (fold change: 58.71047078). Song and colleagues [74] identified 135 lncRNAs which were altered more than two fold. The strongest down-regulated lncRNAs were FER1L4, uc001lsz, BG491697, AF131784, uc009ycs, BG981369, AF147447, HMlincRNA1600 and AK054588, the most up-regulated were H19, HMlincRNA717, BM709340, BQ213083, AK054978 and *DB077273*. Up-regulated H19 in gastic cancer cells accelerates cell proliferation while knockdown of H19 induced apoptosis [44]. The up-regulation of the lncRNA CCAT1, acting as oncogene, was correlated with tumor growth and lymph node metastasis according to Yang *et al.* [43].

The lncRNA HOTAIR was up-regulated in gastric cancer tissue and correlated with larger tumor size, advanced pathological stage, distant metastasis, lymph node metastasis and inferior cell differentiation. Patients with high HOTAIR expression had poorer prognosis concerning the overall survival than those with low levels of HOTAIR. Knockdown of HOTAIR in a murine xenograft model lead to a decrease in tumor volume and tumor weight. The level of HOTAIR expression was associated with the *in vivo* proliferation capacity of cancer cells [42].

Hepatocellular Carcinoma

H19 is exclusively expressed from the maternal allele and plays a key role in genomic imprinting. Still its function remains to be unclear [7]. H19 is upregulated in hepatitis B virus (HBV)-associated hepatocellular carcinoma (HCC) [75]. Lizuka *et al.* [76] described an imbalance of IGF2 expression levels and H19 transcripts and the positive correlation with the progression of HCC. The *H19* gene was reported to interact with drug resistant liver cancer cells by regulating multi drug resistance 1 (MDR1) promoter methylation and induction of P-glycoprotein expression [77]. Overexpression of H19 mRNA and MDR1 was observed in doxorubicin-resistant R-HepG2 cells whereas knockdown of H19 in these cells suppressed MDR1/P-glycoprotein expression and sensitized the cells to doxorubicin toxicity [77]. E-cadherine (CDH1), keratin-8 (KRT-8), keratin-19 (KRT-19) and claudin 1 (CLDN1), the markers for epithelial-to-mesenchymal transition, can be decreased by H19 and suppress HCC progression [45]. Highly up regulated in liver cancer (HULC) is situated at chromosome 6p24.3 and is upregulated in HCC cells. HULC can be used as HCC biomarker as it was detected in plasma [47]. A correlation between HULC and hepatitis B virus X protein (HBx) was obsereved in hepatoma G2 cells and immortalized normal liver L-02 cells. HBx could upregulate HULC and promoted proliferation of hepatoma cells by suppressing p18 [48]. The LncRNA Dreh (HBx-related long non-coding RNA) can repress the expression of vimentin and change the normal cytoskeleton structure. It is downregulated by HBx and acts as a tumor suppressor in the HBV-HCC and inhibit the growth and metastasis *in vitro* and *in vivo* [49].

HOTAIR is also overexpressed in HCC and hepatoma cell lines. Knockdown of HOTAIR reduces the proliferation of the HCC cell line Bel7402 by suppressing matrix metalloproteinase-9 and vascular endothelial growth factor. Higher levels of HOTAIR were detected in patients with lymph node metastasis than in patients without [46]. The lncRNA maternally expressed gene 3 (MEG3) is involved in HCC progression and its expression is reduced in HCC tissue and cell lines compared to normal liver tissue. Braconi *et al.* [50] inducted MEG3 expression in HCC tissue and observed a decrease in cell growth and induced apoptosis.

Yuan *et al.* [51] identified the lncRNA-associated microvascular invasion in HCC (lncRNA MVIH), which was associated with angiogenesis of HCC. The group could promote tumor growth and intrahepatic metastasis *in vivo* and *in vitro* by inhibiting phosphoglycerate kinase 1 (PGK1) secretion. Mineral dust-induced gene (MDIG), another lncRNA, is also overexpressed in HCC [52]. Ogasawara *et al.* [78] detected that MDIG expression levels in surgically resected HCC were higher in tumors larger than 2 cm than in smaller ones. In poorly differentiated HCC the expression was higher than in well differentiated ones.

## 2. Colorectal Cancer

Colorectal cancer (CRC) is the third most commonly diagnosed cancer worldwide with an increasing incidence in men and women [79]. At early tumor stage, the five-year survival rate ranges from 60%–95% whereas in tumors at higher tumor stages the number decreases to just 35% [80,81]. Despite many studies on the pathogenesis of CRC, a unique scheme for CRC development could not be developed [82].

### 2.1. Clinical Presentation

CRC patients can be separated in non-symptomatic and symptomatic individuals. Many of the first group are diagnosed based on a positive colonoscopy result following a positive fecal occult blood test. Symptomatic patients often suffer from rectal bleeding, abdominal pain, changes in bowel habits, anemia and occult bleeding [83]. Due to the anatomical location, the symptoms can vary. Haematochezia and obstructive symptoms are more common for left sided tumors whereas patients with right sided tumors present more often with anaemia without visible haematochezia [84]. Patients can be admitted as emergency with obstructive symptoms like ileus or with signs of acute abdomen in case of tumor perforation. CRC is staged on the basis of the current issue of the TNM classification (classification of malignant tumors) [85].

### 2.2. Subtypes

CRC can either be inherited or sporadic. Currently, 10%–15% of all CRC cases are inherited diseases. Hereditary nonpolyposis colorectal cancer (HNPCC) and familial adenomatous polyposis (FAP) are the most commonly CRC associated genetic disorders [86]. Clinical and familial criteria have been defined to identify patients with HNPCC-associated CRC. The Amsterdam II Criteria and Revised Bethesda Guidelines are routinely used [87,88]. Testing for microsatellite instability (MSI) and DNA mismatch repair genes (MMR) are obligatory if the mentioned pre-selection criteria are rated positive. HNPCC syndrome is caused by an autosomal dominant genetic mutation in one of the four MMR genes—*MLH1*, *MSH2*, *MSH6*, *PMS2*. DNA repair defects increase the number of somatic mutations. Carriers of a MMR gene mutation have a high risk to develop CRC [89].

Patients with FAP syndrom suffer from a germline mutation of the adenomatous polyposis coli (*APC*) gene located on chromosome 5q21 following an autosomal dominant inheritance. “*De novo*” mutations have been detected in 15%–20% of patients without family history [90]. The development of hundreds of adenomas in the colon and rectum, and extracolonic manifestations are the clinical criteria for diagnosing FAP and indicate genetic analysis. Almost 100% of FAP patients will develop CRC by the mean age of 40–50 years if not treated surgically at an early stage [91].

### 2.3. Tumor Markers

Carcinoembryonic antigen (CEA) levels may serve as biomarker during follow-up, but cannot be used as reliable screening or diagnostic tool because of a lack in sensitivity and specificity in the early detection of CRC [92,93,94,95]. Rising CEA levels in the follow-up period after surgery may indicate recurrent disease and the patient has to be restaged through computer tomography (CT) scan of the liver [96].

### 2.4. Staging and Therapy

Before surgery, abdominal ultrasound, colonoscopy, thoracic X-ray and CEA blood levels are routinely analyzed. In case of suspected distant metastasis extended CT scans (brain, abdomen) are recommended. In rectal cancer, patients additional magnetic resonance imaging (MRI) of the pelvis and transrectal endoscopic ultrasound are obligatory. The rectal location has to be evaluated by non-flexible endoscopy as well.

Based on the TNM classification, local *versus* radical, curative *versus* palliative surgery ranging from local excision to pelvic exenteration has to be performed. Preoperative radio-chemotherapy of rectal carcinomas and postoperative chemotherapy of patients with pT3-4 or N+ stage are part of the treatment options supplementing surgery [97]. Recently, many molecular markers including single nucleotide polymorphisms in protein-coding genes, inflammatory scores or microRNAs have been proposed as pathophysiological and prognostic factors in CRC [98,99,100].

### 2.5. LncRNAs in Colorectal Cancer

**Figure 1:**
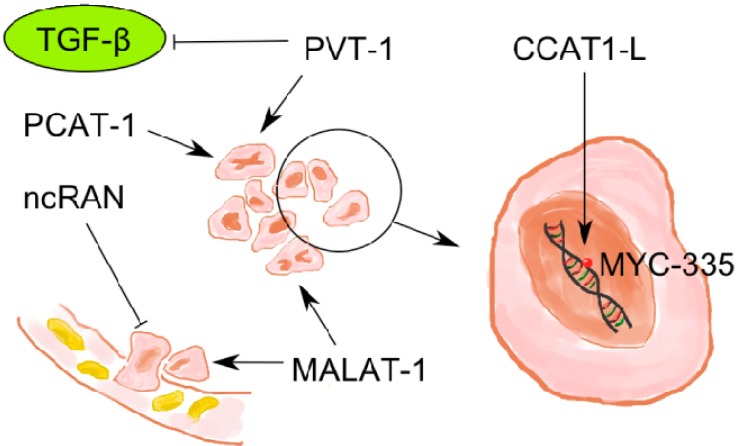
LncRNAs being involved in molecular pathogenesis of colorectal cancer: lncRNAs interact with specific molecular structures, induce different effects in tumor cells and influence the molecular pathogenesis of colorectal cancer.

As mentioned above, recent investigations on lncRNAs have revealed their important roles in genesis and progression of various tumors. Similarly, in colorectal cancer many lncRNAs have been detected as potential biomarkers. Additionally, Figure 1 illustrates the functions of lncRNAs in CRC (Figure 1).

#### 2.5.1. MALAT-1

One of the most prominent lncRNAs is metastasis-associated lung adenocarcinoma transcript 1 (MALAT-1; also known as HCN, NEAT2, NCRNA00 047 and PRO2853), located on chromosome 11q13.1 with a length of 8000 nt [30,55]. It was revealed that MALAT-1 is highly expressed in metastases of various tumors, such as non-small cell lung cancer, hepatocellular carcinoma and endometrial stromal sarcoma [30]. MALAT-1 can be subdivided into 5 fragments that are overexpressed in CRC tissue, all having different functions [55]. One of these fragments, 6918–8441 nt, enhances cell proliferation and invasion, while fragement 5434–6951 nt is important for normal cell function [55]. Therefore, up-regulation of fragment 6918–8411 nt in CRC tissue leads to increased tumor growth. An additional mutation in fragment 5434–6951 nt might promote tumorigenesis in CRC tissue [55].

#### 2.5.2. PVT-1

The oncogenic lncRNA PVT-1 is up-regulated in CRC in comparison to normal colorectal tissue due to a copy number amplification of chromosome 8q24 on which the *PVT-1* gene is located [58]. High levels of PVT-1 in CRC cells lead to increased tumor growth whereas knockdown activates genes of the TGF (transforming growth factor)-β family [58]. Patients suffering from CRC with extremely high levels of PVT-1 presented with greater venous invasion and larger size of lymph nodes in comparison to CRC patients with a lower rate of PVT-1 [58].

#### 2.5.3. ncRAN

ncRAN, at first discovered as a lncRNA up-regulated in neuroblastoma indicating a poor prognosis, also plays a role in CRC [27,28]. According to Qi *et al.* [28] ncRAN is generally under-expressed in CRC in comparison to adjacent tissue. ncRAN inhibits CRC cell invasion and migration but does not affect CRC cell proliferation. Furthermore, it was discovered that CRC with poor differentiation have significantly lower ncRAN levels compared to highly differentiated CRC. Regarding overall survival of patients with CRC, the expression of ncRAN appears to be an important marker [28]. Moreover, in case of ncRAN, it is evident that lncRNAs have distinctive expression patterns in different tumors [101].

#### 2.5.4. CCAT1-L

In CRC the lncRNA colorectal cancer associated transcript-1, long isoform (CCAT1-L), located on chromosome 8q24 shows upregulation which is positively related to tumor stage and progression [56,57]. According to Xiang and colleagues [57,102] the long isoform of CCAT1 (CCAT1-L) operates in the nucleus while the short isoform (CCAT1-S) is found in the cytoplasm. CCAT1-L is located on the MYC (myelocytomatosis)-locus 515 kb distant from MYC but it can interact with a transcriptional enhancer (MYC-335) by chromatine looping which subsequently interacts with the MYC-promotor [103]. Furthermore, knockdown of CCAT1-L leads to a decreased level of MYC mRNA, emphasising the theory that this lncRNA can regulate MYC-expression *in cis* [57].

#### 2.5.5. CCAT2

Very recently, Ling *et al.* [104] reported the novel lncRNA colon cancer associated transcript 2 (CCAT2) encompassing the rs6983267 SNP (single nucleotide polymorphism) which is highly overexpressed in microsatellite-stable colorectal cancer and promotes tumor growth, metastasis, and chromosomal instability. Furthermore, they showed interaction with Wnt signaling, an important signal transduction pathway in CRC. These findings were corroborated by a breast cancer study in in which the same group showed an association of CCAT2 with aggressive biological behavior [105]. In lung cancer, CCAT2 is up-regulated in NSCLC tissue and its expression level is associated with invasive potential [106].

#### 2.5.6. PCAT-1

Prostate cancer-associated ncRNA transcripts 1 (PCAT-1) was reported to be correlated with poor prognosis in CRC patients [53]. The PCAT-1 gene is located on chromosome 8q24 [37]. Ge *et al.* [53] identified PCAT-1 overexpression in CRC tissue as an independent predictor of poor overall survival time in a cohort of patients. Distant metastases were positively associated with PCAT-1 overexpression of in CRC tumor cells. In prostate cancer PCAT-1 overexpression increased cell proliferation *in vitro* promoted by PRC2 although the underlying molecular mechanism in CRC is still unclear [37].

#### 2.5.7. HOTAIR

The lncRNA HOTAIR was reported to be overexpressed in CRC. High expression levels were associated with poorer overall survival in the study cohort and were linked to liver metastasis. *In vitro* the overexpression increased the invasiveness of CRC cells [59].

#### 2.5.8. PRNCR1

The association between polypmorphisms in prostate cancer non-coding RNA 1 (PRNCR1) on chromosome 8q24 and the risk of CRC was reported by Li and colleagues [54] who investigated five single nucleotide polymorphisms (SNPs). The alleles *rs1456315G* and *rs13252298* were associated with increased tumor size and a decreased risk of developing CRC. The alleles *rs7007694C* and *rs16901946G* were reported to be associated with a decreased risk to develop poorly differentiated CRC while *rs1456315G* was associated with an increased risk. Due to this data SNPs in the lncRNA PRNCR1 may be related to the development of CRC [54].

## 3. Discussion

The detection of lncRNAs in the early 21st century opened up new possibilities in cancer research [1]. LncRNAs are larger than 200 nucleotides and can reach sizes of 100 kilobases [4]. As lncRNAs are not translated into proteins, their regulative functions can take place faster in comparison to proteins [14]. Various studies over the past few years have revealed the important role of lncRNAs in tumorigenesis [24].

The molecular mechanisms of some lncRNAs as well as their roles in different tumors have been very well established, however, intensive investigations on prominent lncRNAs such as MALAT-1 point out the fact, that lncRNAs have tissue specific purposes [30,41,60]. 

They can act either as oncogenes or tumorsuppressor-genes although some lncRNAs act like oncogenes in one tumor while displaying suppressory functions in another (Table 2). TUG-1 for example is underexpressed in non-small cell lung cancer while overexpression can be found in osteosarcoma cells compared to control tissue [29,31].

In addition, in colorectal cancer different lncRNAs have been detected as potential pathogenesis-driving factors. MALAT-1, HOTAIR, PVT-1 and CCAT1-L are up-regulated in colorectal cancer tissue while ncRAN is down-regulated [28,55,56,57,58,59]. However, some lncRNAs consist of several fragments, each of them with different functions [55].

In summary, lncRNAs seem to play an important and previously underestimated role in human cancer. Although for some of them a functional characterication has been successfully performed, the yet identified role of lncRNAs in human cancer seems rather to be the tip on the iceberg than anything else. Therefore, further investigations will be necessary to understand the numerous molecular mechanisms performed by lncRNAs in human cancer.

**Table 2:** LncRNAs in Cancer.

Name	Function	Cancer	Expression	Ref.
LINC00312	cell-cycle inhibition	nasopharyngeal carcinoma	down	[25]
PlncRNA-1	binds to androgene-receptor	prostate cancer	up	[34]
	suppressor of apoptosis	esophagus squamous cell carcinoma	up	[40]
HOTAIR	proliferation, invasion	CRC (colorectal cancer)	up	[59]
		HCC (hepatocellular carcinoma)	up	[46]
	-	gastric cancer	up	[42]
	-	esophagus squamous cell carcinoma	up	[41]
	-	epithelial ovarian cancer (EOC)	up	[38]
ASLNC22381	targeting IGF-1 genes	glioblastoma multiforme	up	[26]
ASLNC20819	targeting IGF-1 genes	glioblastoma multiforme	up	[26]
TUG1	epigenetically regulating expression of HOXB7	non-small cell lung cancer	down	[29]
	-	osteosarcoma	up	[29,31]
PVT-1	suppressor of TGF (transforming growth factor) β	CRC	up	[58]
ncRAN	inhibits cell migration and invasion	CRC	down	[27,58]
	-	urothelial carcinoma	up	[32]
	-	neuroblastoma	up	[27,28]
CCAT1-L	MYC-335 (enhancer) interaction	gastric cancer	up	[43]
	-	CRC	up	[56,57]
MALAT-1	invasion, progression	CRC	up	[55]
	-	HCC	up	[30]
	-	lung cancer	up	[30]
	-	prostate cancer	up	[35]
	suppressory role	EOC	down	[39]
H19	interacts with E-cadherin, keratin 8 + 19, claudin-1; proliferation	HCC	up	[45]
	-	gastric cancer	up	[44]
	suppressory role	EOC	down	[39]
UCA-1	proliferation, migration, invasion	EOC	up	[39]
	-	urothelial carcinoma	up	[33]
PCGEM1	bind to androgen receptor (AR), transcriptation	prostate cancer	up	[36]
	-	CRC	up	[54]
PRNCR1	bind to AR, transcriptation	prostate cancer	up	[36]
PCAT-1	-	CRC	up	[53]
	proliferation, suppression of BRCA2 tumor suppression gene	prostate cancer	up	[37]
HULC	suppressing p18, proliferation	HCC	up	[47,48]
LncRNA-Dreh	inhibit growth and metastasis	hepatitis B virus-HCC	down	[49]
MEG3	inducing cell apoptosis	HCC	down	[50]
lncRNA-MVIH	invasion, angiogenesis	HCC	up	[51]
MDIG	histological grading	HCC	up	[52]

## 4. Conclusions

Different lncRNAs involved in various human diseases have been investigated in recent years. As figured out in this article, these specific RNA transcripts can effectively influence molecular and cellular processes without being translated into proteins. In tumor cells, particular lncRNAs can be either up-regulated or down-regulated, resulting in significant changes of molecular pathogenesis and clinical stage.

Moreover, lncRNAs show a tissue specific up- or down-regulation, leading to the expectation that lncRNAs may represent new targets in tumor therapy.

In colorectal cancer, at least 8 lncRNAs show discrepancies in cellular regulation, though the very same lncRNA could be regulated conversely when examined in other tumor tissues.

Therefore, the meaning and function of lncRNAs has to be further investigated, as a single lncRNA often has more than only one feature in human cells. Furthermore, a consistent nomenclature of recently detected lncRNAs may facilitate the researcher’s work.
